# Fluorescent Protein Solid‐State Luminescent Solar Concentrators

**DOI:** 10.1002/smll.202507761

**Published:** 2025-10-22

**Authors:** Sihan Lei, Sara Ferrara, Sanchari Chowdhury, Ruben D. Costa

**Affiliations:** ^1^ Technical University of Munich, Campus Straubing for Biotechnology and Sustainability Chair of Biogenic Functional Materials Schulgasse 22 94315 Straubing Germany

**Keywords:** fluorescent proteins, light‐guiding matrix, luminescent solar concentrator, protein‐epoxy stabilization, solar window

## Abstract

Building integration of Silicon‐photovoltaic (Si‐PV) panels into urban infrastructure faces efficiency and aesthetic concerns. An effective alternative is the luminescent solar concentrators (LSCs), in which the emitters embedded in polymer blocks capture, converts, and guide the sun irradiation to the edges where Si‐PV panels are placed. The best LSCs are based on rare‐earth and/or toxic emitters, which undermines the sustainability of this technology. In this context, a few groups have implemented biogenic emitters like fluorescent proteins (FPs) and protein systems in liquid‐state LSCs, reaching the maximum optical efficiency (η_opt_) of 6.88%. However, liquid‐state LSCs face low photostability and easy leakage issues. Herein, it is demonstrated that the concept of FP solid‐state LSCs, integrating an archetypal FP (T‐Sapphire) in light‐guiding epoxy materials of arbitrary shapes. They are optimized with respect to the water content and the type/amount of stabilizers, fairly keeping the photoluminescence features over 250 days under storage conditions. This allows the fabrication of FP solid‐state LSCs that achieve a 32‐fold enhanced stability compared to liquid‐state LSCs (8 days vs 6 h) and the best η_opt_ of 7.41%. Overall, this work provides a key advancement toward truly sustainable LSCs, marking a major step toward biologized photovoltaics.

## Graphical abstract

This work demonstrates the concept of ultilizing Fluorescent proteins (FPs) as the sustainable emitters for of solid‐state luminscent solar concentrators (LSCs). By integrating T‐Sapphire into a light‐guiding polymer epoxy, the devices achieved enhanced performance compared with liquid‐state FP‐based LSCs.

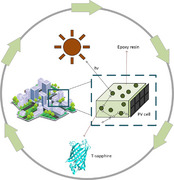

## 1 Introduction

The implementation of zero‐energy consumption buildings, in general, and the integration of solar‐harvesting components into urban infrastructures, in particular, are playing an instrumental role in the contemporary architecture to enable the sustainable transformation of our society. However, the best Silicon‐photovoltaic (Si‐PV) panels are not optimal as integrated energy‐harvesting architectural components. This is related to i) their significantly reduced performance under shading conditions, and ii) the lack of color aesthetic that is essential for design‐sensitive applications.^[^ ^1^ ^]^ This has fueled the interest in revisiting the luminescent solar concentrator (LSC) concept over the last decade.^[^ ^2^, ^3^ ^]^

LSCs consist of a light‐guiding polymer coating doped with organic/inorganic emitters that can collect, down‐convert, and redirect direct and/or diffuse sun radiation into any type of solar cells placed at the edges of the polymer coating.^[^ ^4^, ^5^, ^6^, ^7^, ^8^ ^]^ Regarding the choice of wave‐guiding polymer,^[^ ^9^ ^]^ polymethylmethacrylate (PMMA) is currently the most used material in LSCs, while epoxy resins are attracting more attention as they can be prepared at room temperature are derived from renewable resources,^[^ ^10^, ^11^ ^]^ and show suited optical features: a very high transmittance (90% at 400–800 nm range)^[^ ^12^ ^]^ and a refractive index of 1.5–1.56 (higher than PMMA 1.49),^[^ ^13^ ^]^ making it suitable for application in FP‐based LSCs. Regarding emitters, we could classify them into two types: i) inorganic, such as rare‐earth ions,^[^ ^14^, ^15^, ^16^ ^]^ metal complexes,^[^ ^17^, ^18^ ^]^ quantum dots (QDs),^[^ ^19^, ^20^, ^21^, ^22^, ^23^ ^]^ nanocrystals,^[^ ^24^, ^25^, ^26^ ^]^ etc., that have resulted in LSCs with the maximum optical efficiency (η_opt_) of 7.9%,^[^ ^27^ ^]^ and ii) organic, such as dyes^[^ ^28^, ^29^ ^]^ and polymers,^[^ ^30^, ^31^ ^]^ which have led to LSCs with the η_opt_ of 10.4% at the G of 25—Table S1 (Supporting Information).^[^ ^32^ ^]^

**Figure 1:**
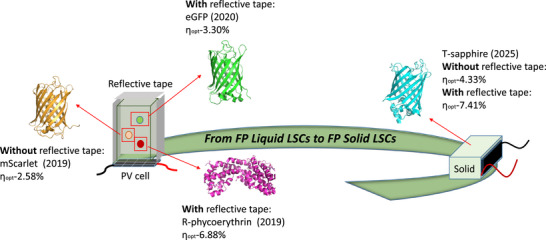
Schematic representation of the state‐of‐the‐art protein‐based liquid‐state LSCs (left) and the concept of protein‐based solid‐state LSCs (right), highlighting the type of protein and main LSC figures‐of‐merit.

A common feature of the above state‐of‐the‐art LSC is the use of rare‐earth, toxic, and/or expensive emitters, which hinders its transition to becoming a truly sustainable technology. Thus, the biologization of LSCs with respect to integrating biogenic emitters and hosts is an emerging frontier in the field. To date, there is a handful number of examples, in which proteins have been integrated in liquid‐state LSCs—i.e., aqueous buffer solutions with R‐phycoerythrin (R‐PE),^[^ ^33^ ^]^ enhanced green fluorescent proteins (eGFP),^[^ ^34^, ^35^ ^]^ and mScarlett,^[^ ^36^ ^]^ placed in a glass/polymer reservoir, whose six edges are typically covered by four external reflecting coatings and one solar cell—**Figure** **1** and Table S1 (Supporting Information). These protein‐based liquid‐state LSCs featured competitive η_opt_ values up to 6.88%.^[^ ^33^ ^]^ Unfortunately, none of the above studies discloses the stability of these protein‐based liquid‐state LSCs under either ambient storage or operating conditions.

Herein, we put forward the concept of fluorescent protein (FP) based solid‐state LSCs—Figure 1, demonstrating three important aspects. First, we provide the optimized procedure to integrate archetypal FPs like T‐Sapphire^[^ ^37^ ^]^ into a commercial light‐guiding epoxy resin with respect to the amount of water as well as the type of branched polymer stabilizer to preserve their photoluminescence features over time—i.e., fairly no loss in photoluminescence quantum yields (ϕ) going from aqueous solution to epoxy materials, and somewhat keeping the ϕ values for >250 days upon storage conditions. Second, the FP‐based solid‐state LSCs exhibited a 32‐fold higher stability than traditional FP‐based liquid‐state LSCs under same operation conditions (8 days vs 6 h). Third, the best T‐Sapphire‐epoxy based solid‐state LSCs (G of 2.9) resulted in η_opt_/photon‐to‐current conversion efficiency (PCE) values of 7.41%/0.75% and 4.33%/0.41% using a single Si‐PV panel with and without applying reflecting coatings, respectively.

In light of our findings, the headline of this work is that the concept of FP‐based solid‐state LSCs might provide an effective and simple method for integrating FPs into epoxy matrices with competitive performance. Hence, this novel approach fosters the biological transformation toward sustainable LSCs.

## 2 Results and Discussion

### 2.1 FP‐Epoxy Coatings for LSCs

**Figure 2:**
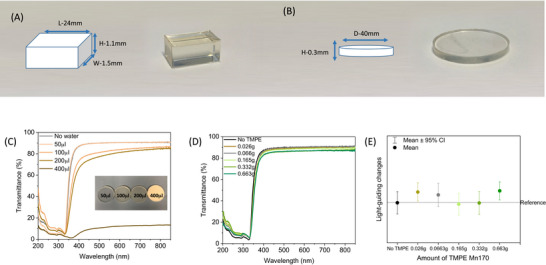
Sketches of different shapes and sizes of A) epoxy block and B) disk. Changes of the transmittance spectra (C) upon increasing the amount of PBS buffer solution in the epoxy disk (see legend) with a picture of epoxy disks as inset. Changes of D) the transmittance spectra and E) light‐guiding features upon increasing the amount of TMPE‐170 in the epoxy disk prepared with 50 µL PBS buffer (see legends).

Similar to what has been described in the literature,^[^ ^38^, ^39^, ^40^, ^41^ ^]^ the implementation and stabilization of FPs in hydrophobic polymer matrices typically requires small amounts of aqueous solutions and stabilizers that might not be compatible with epoxy matrices in terms of curing and optical features. In this work, we selected the commercial epoxy resin that cures at room temperature upon mixing both components to form arbitrary shapes—**Figure** **2**A,B and experimental section for details. As shown in Figure 2C, the amount of aqueous PBS buffer solution (pH 7.4, Phosphate‐buffered saline—see Experimental Section) must be kept below 50 µL to avoid visual opaqueness. This is also confirmed by the loss of the transmittance features of, for example, the epoxy disks upon increasing the amount of PBS buffer solution—Figure 2C. Next, we studied the impact of using branched polymer stabilizers such as trymethylpropane ethoxylate (TMPE; M_n_ of 170, 450, and 1014) that are known to provide a better compatibility environment surrounding the FPs in dry polymer environments.^[^ ^42^, ^43^, ^44^ ^]^ Here, TMPE derivatives can sustain the hydrogen bonding network at the protein surface. This helps to preserve the protein structure within the polymer matrix as well as impacts on the typical degradative pathways, such as oxidation, H‐assisted processes, and/or isomerization.^[^ ^44^, ^45^, ^46^, ^47^ ^]^ In detail, we focused on how the amount of each stabilizer impacts on both, the optical features of the epoxy and the stabilization of the FPs—*vide infra*. As an example, the transmittance of the epoxy disks is reduced using >0.066 g of TMPE‐170—Figure 2D, while the light‐guiding holds fairly constant, regardless of the amount of TMPE‐170 with respect to the reference neat epoxy material—Figure 2E. These findings are the same for the other TMPE derivatives—Figure S1 (Supporting Information). Finally, the best epoxy blocks (50 µL PBS buffer with 0.066 g TMPE‐170) exhibited a very weak emission band centered at 410 nm with a ϕ of 2% and an average excited state lifetime (<τ>) of 5.03 ns—Figure S2 (Supporting Information).

**Figure 3:**
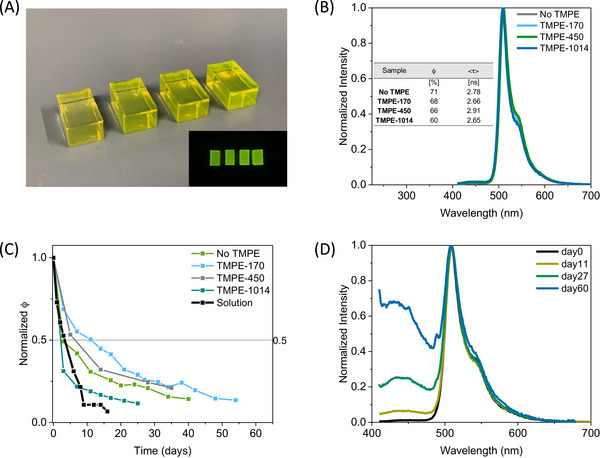
T‐Sapphire‐epoxy blocks (A) under ambient (top) and UV excitation (bottom). Emission spectra of T‐Sapphire‐epoxy with different TMPE derivatives ((B) see legend) with the inset table gathering the figures‐of‐merit of ϕ (λ_ex_ = 400 nm) and <τ> (λ_ex_ = 375 nm). Normalized ϕ (λ_ex_ = 400 nm) decay profile of both solution and T‐Sapphire‐epoxy samples ((C) see legend) under white LED simulated one sun irradiation over time. Emission spectra changes of T‐Sapphire‐TMPE‐170‐epoxy samples (D; see legend) under white LED simulated one sun irradiation over time.

In stark contrast to the optical features, the type of TMPE is key for the stabilization of FPs in the epoxy matrices. In short, we selected the T‐Sapphire, since it is a large Stokes shift FP with a green emission band centered at 508 nm associated to a ϕ of *ca*. 65% and <τ> of 3.36 ns in PBS buffer solution—Figure S3 (Supporting Information).^[^ ^37^ ^]^ As a reference, a 50 µL PBS solution containing the same amount of T‐Sapphire with/without TMPE derivatives (i.e., 1:1000 FP:TMPE mol ratio) was mixed with the epoxy upon gentle stirring without noting any color change and/or bubble formation to the naked eye. This is immediately followed by adding the hardener upon soft stirring and then poured to the desired mold shape for 48 h at ambient conditions—see Experimental Section for details. All the T‐Sapphire‐epoxy blocks exhibited a green emission band centered at 511 nm with ϕ and <τ> values of around 66% and 2.8 ns—**Figure** **3**A,B. However, their photostability under white LED simulated one sun irradiation is significantly dependent to the presence and the type of polymer stabilizer—Figure 3C. In short, T‐Sapphire‐epoxy blocks without any stabilizer showed a quick reduction of their ϕ (50% loss) after 3 days, while the use of TMPE stabilizers increases the photostability, reaching the best values of 11 days with TMPE‐170 derivative—Figure 3C. For reference purposes, the photostability of T‐Sapphire in PBS solution was also investigated under the same conditions, reaching 50% of the initial ϕ value after 3 days—Figure 3C. What is more, T‐Sapphire‐epoxy blocks with TMPE‐170 derivative maintained 70% of its fresh ϕ after 250 days upon storage conditions—Figure S3 (Supporting Information). Hence, the combination of FP‐TMPE‐170 epoxy stands out among the use of other stabilizers (TMPE‐450/‐1014) as well as those without stabilizers. This may be attributed to the relatively larger size of these TMPE derivatives, which could result in the formation of large disorder hydrophilic domains. Here, the ability to effectively exclude water from the protein surface, while preserving its structure could be compromised. In addition, large polymer chains could induce perturbations in the local environment of the protein surface, thereby reducing its compatibility within the polymer matrix.^[^ ^48^ ^]^

In order to understand the degradation mechanism, the emission band changes of the T‐Sapphire‐TMPE‐170‐epoxy blocks were monitored over time—Figure 3D. In detail, the T‐Sapphire emission band intensity reduces, while a new emission band located at 410 nm slowly evolves. The latter could be attributed to the poorly emissive protonated form of the chromophore^[^ ^49^, ^50^, ^51^ ^]^ and/or the emission of the epoxy − *vide supra*. Unfortunately, the intense absorbance of the epoxy at <300 nm—Figure S2 (Supporting Information)—does not allow a direct excitation of the tryptophan residues, hampering a qualitative check about the partial distortion of the protein scaffold.^[^ ^49^, ^50^, ^51^ ^]^ Thus, we closely inspect the τ values of the emission bands centered at 510 nm at 375 nm excitation over time. In short, the fresh τ values of the emission at 510 nm obeys a bi‐exponential fitting with a high‐predominant exponential factor for a τ value of 2.1 ns and a low‐predominant exponential factor for a long τ of 3.6 ns. Over time, the first component progressively reduces down to 2.1/1.8/1.4 ns (11/27/60 days) and loses weight—Table S2 (Supporting Information), suggesting that the chromophore of T‐Sapphire is deactivated. This could include the transformation to the less‐emissive neutral form, *cis*/*trans* isomerization, and/or oxidation processes.^[^ ^43^, ^51^, ^52^, ^53^ ^]^ In parallel, the second component increases in prevalence, reaching values of 4.5/5.0/5.2 ns (11/27/60 days) that could be attributed to the τ values of the epoxy emission – *vide supra*. Indeed, the epoxy emission matches with the absorption of the T‐Sapphire—Figure S3 (Supporting Information), suggesting a possible energy transfer between both components. These trends are also valid for the other T‐Sapphire‐epoxy blocks with different stabilizers—Table S2 (Supporting Information).

### 2.2 FP‐Based Solid‐State LSCs

**Figure 4:**
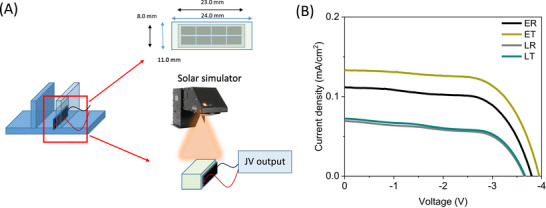
A) Scheme of LSCs coupled with a Si‐PV panel and the measuring strategy. B) Current density‐voltage of reference and T‐Sapphire solid‐ and liquid‐based LSCs (see legend).

To provide a fair comparison within the different LSC architectures, we fabricated LSCs with T‐Sapphire‐TMPE‐170‐epoxy and T‐Sapphire PBS solution as targets (**E‐T** and **L‐T**) with respect to those FP‐free epoxy (50 µL PBS buffer with 0.066 g TMPE‐170) and PBS solution as references (**E‐R** and **L‐R**). Here, the LSCs were finalized with one commercial Si‐PV panel (IXOLAR^TM^) placed at the edge of either the epoxy (**E‐T**/**R**) or quartz cuvette (**L‐T**/**R**)—Figures 1 and **4**. The scheme for the current density‐voltage (*J–V*) and incident to photon‐to‐current conversion (IPCE) measurements is shown in **Figure** 4A, while **Table** **1** summarizes all the figures‐of‐merit of the LSCs under 1 sun AM 1.5 illumination conditions—see Experimental Section for details.

**Table 1:** Figures‐of‐merit of both FP‐based liquid and solid‐state LSCs. Mean ± s.d.; n = 3 independent experiments.

	J_sc_ ^a)^	V_oc_ ^b)^	FF^c)^	η_opt_ ^d)^	PCE^e)^	η_opt_ /PCE gains^f)^	Estimated J_sc_ ^g)^
	[mA cm^−2^]	[V]	[%]	[%]	[%]	[%]	[mA cm^−2^]
**L‐R**	0.068 ± 0.001	3.60 ± 0.09	60 ± 0	1.35 ± 0.03	0.136 ± 0.002	—	—
**L‐T**	0.072 ± 0.004	3.60 ± 0.07	62 ± 2	1.41 ± 0.01	0.145 ± 0.006	4	—
**E‐R**	0.112 ± 0.003	3.83 ± 0.03	63 ± 2	3.12 ± 0.08	0.276 ± 0.008	—	0.101
**E‐T**	0.132 ± 0.002	3.92 ± 0.03	64 ± 1	3.60 ± 0.06	0.333 ± 0.009	15/18	0.121

^a)^ Short‐circuit current density;

^b)^ Open‐circuit voltage;

^c)^ Fill Factor;

^d)^ Optical efficiency;

^e)^ Photon conversion efficiency;

^f)^ Relative gain of η_opt_ and PCE in LSC‐PV systems with respect to the reference **L‐R** and **E‐R**;

^g)^ Short‐circuit current density calculated from the IPCE measurements;

All the LSCs featured the expected light harvesting efficiency with a *J–V* curve response with a similar fill factor (FF) of 60–64%, but with a higher open‐circuit voltage (V_oc_) and short‐circuit current density (J_sc_) for the solid‐state LSCs compared to those of liquid LSCs as widely noted in the literature—Figure 4B and Table 1.^[^ ^54^, ^55^, ^56^ ^]^ Indeed, **E‐T** LSCs exhibit superior relative gain with respect to the η_opt_ and PCE of **E‐R** with a value of 15% and 18%, respectively. In addition, the estimated J_sc_ from the IPCE is in line with the device measured value—Table 1. More importantly, the IPCE response nicely shows the contribution of T‐Sapphire enhancing the light‐harvesting capability of the Si‐PV panel in the high‐energy region of the visible spectrum without affecting the response in the low‐energy region with respect to the reference—Figure S4 (Supporting Information). Finally, the FP solid‐state LSCs (G = 2.9) already feature a remarkable η_opt_ = 3.60% compared to the prior‐art biogenic emitter‐based LSCs—Figure 1.^[^ ^33^, ^34^, ^36^ ^]^ Although the calculation of the η_opt_ must be considered qualitative due to its neglect of the spectral response of the edge‐mounted Si‐PV panel, it is necessary for the comparison with previous FP‐based LSCs. For example, the liquid‐LSCs with eGFP (30‐fold amount higher than our work) and four reflecting coatings featured η_opt_ = 3.3% with G = 2,^[^ ^34^ ^]^ while chlorophyll (27‐fold amount higher than that used in this work) solid‐state LSCs without reflecting coatings showed a η_opt_ = 3.7% with G = 3.3.^[^ ^57^ ^]^ Hence, we proceed to further optimize our FP solid‐state LSCs with regards to T‐Sapphire concentration and use of reflecting coatings.

### 2.3 Optimizing FP‐Based Solid‐State LSCs

We started the optimization of T‐Sapphire solid‐state LSCs by increasing the amount of FP going from 0.5 mg up to 4 mg in the epoxy—see Experimental Section for details. The upper limit was dictated by the strong aggregation of the highly concentrated protein solution. Indeed, the emission band of T‐Sapphire‐TMPE‐170‐epoxy blocks is slightly red‐shifted upon increasing amount, indicating a certain degree of agglomeration of protein in the epoxy matrix—Figure S5 (Supporting Information). However, this does not significantly impact the ϕ values—Figure S5 (Supporting Information).

**Figure 5:**
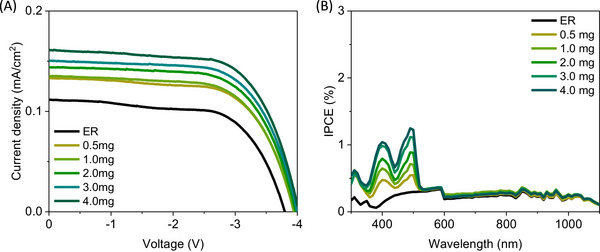
A: Current density‐voltage (A) and IPCE scans (B) of solid‐state LSCs upon increasing the amount of T‐Sapphire (see legend).

For comparison, a Si‐PV panel without any wave‐guiding epoxy was placed vertically (**SV**) to quantify the gain contributed by both, the polymer matrix and the FPs. As expected, the measured J_sc_ and V_oc_ increase from **SV** to **E‐R** due to wave‐guiding properties of the polymer and gradually enhance upon increasing the amount of FPs in the epoxy—**Figure** **5**A and **Table** **2**. In detail, the J_sc_ increases from 0.132 mA cm^−2^ (0.5 mg of T‐Sapphire) to 0.159 mA cm^−2^ (4 mg of T‐Sapphire) and, in turn, the V_oc_ slightly enhances—Table 2. This trend is attributed to the increased light‐harvesting features of the LSCs at the UV–blue region, enabling a more efficient down‐conversion. This was further confirmed by the IPCE response; in which the light‐harvesting capability of the Si‐PV panel in the high‐energy region of the visible spectrum is nicely enhanced upon the increase of T‐Sapphire amount—Figure 5B. Here, the J_sc_ estimated from IPCE measurements shows good agreement with the device‐measured values, as well as with the observed increasing trend—Figure S6 (Supporting Information) and Table 2. Notably, **E‐R** had an 80%/95% gain of η_opt_/PCE with respect to the **SV**. However, the enhanced performance of Si‐PV panels upon using FPs is, therefore, noted by the increase of the relative gain of η_opt_/PCE with respect to **E‐R** from 15%/18% to 39%/46% for 0.5 and 4 mg of T‐Sapphire, respectively—Table 2. This results in the best η_opt_ of 4.33% for LSCs with 4 mg T‐Sapphire.

**Table 2:** Figures‐of‐merit of solid‐state LSCs upon increasing the amount of the T‐Sapphire. Mean ± s.d.; n = 3 independent experiments.

	FP	J_sc_ ^a)^	V_oc_ ^b)^	FF^c)^	η_opt_ ^d)^	PCE^e)^	η_opt_ /PCE gains^f)^	η_opt_ /PCE realative gains^g)^	Estimated J_sc_ ^h)^
	Mg mL^−1^	[mA cm^−2^]	[V]	[%]	[%]	[%]	[%]	[%]	[mA cm^−2^]
**SV**	—	0.064 ± 0.003	3.53 ± 0.02	63 ± 1	1.73 ± 0.07	0.141 ± 0.004	—	—	—
**E‐R**	—	0.112 ± 0.003	3.83 ± 0.03	63 ± 2	3.12 ± 0.08	0.276 ± 0.008	80/95	—	0.101
**E‐T**	0.5	0.132 ± 0.002	3.92 ± 0.03	64 ± 1	3.60 ± 0.06	0.333 ± 0.009	108/136	15/18	0.121
	1	0.135 ± 0.001	3.94 ± 0.05	64 ± 1	3.65 ± 0.04	0.34 ± 0.02	111/141	17/21	0.143
	2	0.146 ± 0.003	3.97 ± 0.05	64 ± 1	3.97 ± 0.09	0.37 ± 0.02	129/162	27/32	0.145
	3	0.150 ± 0.001	3.98 ± 0.05	65 ± 1	4.07 ± 0.02	0.39 ± 0.02	135/176	30/39	0.158
	4	0.159 ± 0.001	4.00 ± 0.06	65 ± 1	4.33 ± 0.04	0.41 ± 0.01	150/191	39/46	0.162
	4.0(**RT**)	0.273 ± 0.001	4.23 ± 0.02	65 ± 0	7.41 ± 0.01	0.746 ± 0.004	328/429	137/168	0.266

^a)^ Short‐circuit current densities;

^b)^ Open‐circuit voltage;

^c)^ Fill Factor;

^d)^ Optical efficiency;

^e)^ Photon conversion efficiency;

^f)^ Gain of η_opt_ and PCE in LSC‐PV system with respect to the reference **SV**;

^g)^ Relative gain of η_opt_ and PCE in LSC‐PV system with respect to the reference **E‐R**;

^h)^ Short‐circuit current density calculated from the IPCE measurements; Reflective tape – **RT**.

As a final optimization step, we applied four reflective tapes to the best performing 4 mg T‐Sapphire ‐TMPE‐170‐epoxy LSCs, as reported in the prior‐art of FP‐based liquid LSCs—Figure 1.^[^ ^33^, ^34^, ^36^ ^]^ This results in a 1.7‐fold increase in η_opt_ compared with no reflective tapes applied, reaching the η_opt_ of 7.41%—Table 2 and Figure S7 (Supporting Information) for *J–V* and IPCE curves. To contextualize our findings, this FP‐based solid‐state LSCs features a remarkable η_opt_ value among LSCs using biogenic emitters—Figure 1,^[^ ^33^, ^34^, ^36^ ^]^ and is comparable to LSCs using conventional emitters.^[^ ^3^, ^27^, ^32^ ^]^ Finally, the device stability was evaluated under continuous white LED simulated one sun irradiation, while monitoring the loss of relative η_opt_ gain over time—Figure S8 (Supporting Information). Here, this figure dropped down to 50% after 8 days under continuous irradiation, resulting in a 32‐fold enhanced stability compared to that of the liquid LSCs. Besides, thermal stress of 40 and 80 °C were also applied—Figure S8 (Supporting Information). Though this stability comparison was carried out with the lowest amount of T‐Sapphire (0.5 mg), this is remarkable compared with the best stability reported to date in LSCs with other biogenic emitters, such as chlorophyll, that is, 40 h for a 1% short current drop.^[^ ^57^ ^]^

## 3 Conclusion

To date, the biologization of LSCs has been largely restricted to aqueous‐based systems incorporating biogenic emitters, such as chlorophyll and fluorescent proteins (FPs), which are prone to leakage and rapid photodegradation.^[^ ^33^, ^34^, ^36^, ^57^ ^]^ Herein, we have demonstrated a fresh approach toward FP solid‐state LSCs successfully bringing together FPs and epoxy resin components, resulting in remarkable η_opt_ of 4.33% and 7.41% without and with reflecting tapes. In detail, we have provided the step‐by‐step optimization for the fabrication of arbitrary shaped FP‐epoxy materials with respect to the amount of water and the amount/type of polymer stabilizers to preserve the resin optical features (transmittance and light‐guiding) and T‐Sapphire emission (efficiency and photostability). Indeed, the best T‐Sapphire‐epoxy materials exhibited a remarkable ϕ value of >65%. Solid‐state LSCs with 0.5 mg T‐Sapphire clearly outperformed the respective liquid LSCs in terms of performance gains (η_opt_/PCE gain of 15/18% vs 4/7%) and the overall device stability (8 days vs 6 h). What is more, these figures are nicely enhanced upon increasing the amount of T‐Sapphire (4 mg) and the use of reflective tapes, reaching, for example, a remakable η_opt_ among the reported biogenic emitters‐based LSCs (7.41% vs 6.88%).^[^ ^33^, ^34^, ^36^, ^57^ ^]^ Overall, this work sets in a novel and effective stepping stone toward the biological transformation of sustainable LSCs, demonstrating easy‐to‐prepare, efficient, and stable FP‐based solid‐state LSCs.

## 4 Experimental Section

### Protein Production and Purification

T‐Sapphire plasmid was purchased from Twist Bioscience and was transformed to Lysogeny broth (LB) plate with kanamycin and expressed in *Escherichia coli* BL21 in Lysogeny broth (LB) culture media from OD 0.4–0.6 for 72 h with 50 µg mL^−1^ final concentration of kanamycin, 1 mm final concentration of inducer IPTG (Isopropyl ß‐D‐1‐thiogalactopyranoside) and 1% final concentration of 80%w/v glycerol. Cells were harvested and sonicated 8 min in total (Amplitude 80, pulse on‐time: 1 s, off‐time 3 s) after expression. Then was purified by HisTrap HP 5 mL column (Cytiva ÄKTA Pure) and desalted using a HiPrep 26/10 Desalting column (Cytiva ÄKTA Pure) in PBS buffer (NaCl 8 mg L^−1^, KCl 0.2 g L^−1^, Na_2_HPO_4_ 1.42 g L^−1^, KH_2_PO_2_ 0.24 g L^−1^, MQ water; pH 7.4). Purified proteins were frozen immediately in liquid nitrogen and stored at ‐80 °C. Before use, thawed proteins were centrifuged at 13.3 g for 30 min at 4 °C, and the supernatant was used for the measurements.

The T‐Sapphire protein sequence is: MVSKGEELFTGVVPILVELDGDVNGHKFSVSGEGEGDATYGKLTLKFICTTGKLPVPWPTLVTTFSYGVMVFARYPDHMKQHDFFKSAMPEGYVQERTIFFKDDGNYKTRAEVKFEGDTLVNRIELKGIDFKEDGNILGHKLEYNFNSHNVYIMADKQKNGIKANFKIRHNIEDGGVQLADHYQQNTPIGDGPVLLPDNHYLSIQSALSKDPNEKRDHMVLLEFVTAAGITLGMDELYKHHHHHH*

### Polymer Matrix Preparation and Characterization

The LSCs were prepared implementing an aliquot of 50 µL PBS solution and constant molar ratio of the protein:TMPE (1:1000) for different trymethylpropane ethoxylate (TMPE) derivatives (M_n_ of 170, 450, and 1014 from Sigma–Aldrich). To prepare a 2.4 cm × 1.5 cm × 1.1 cm block, 3.78 g of DGEBA epoxy (Faserverbundwerkstoffe Company, product number: Epoxy Resin L) was added to the respective aqueous solution, and the resulting mixture was gently stirred, and followed by adding 1.32 g of the hardeners (Faserverbundwerkstoffe Company, product number: Hardener CL) on top upon gentle stirring. The mixture was poured in the desired mold and left to dry at ambient conditions for 48 h. Here, several coatings were produced upon increasing the amount of water and different TMPE derivatives (3 for each sample) with the shape of a 4 cm × 0.32 cm disk. The transmittance was measured by a Shimadzu UV–vis spectrophotometer Uv‐2700i. The light‐guiding property tests were done by irradiation on the main surface with a WINGER WEPRB1‐S1 Power LED Star royal blue (450 nm) powered with Keithley 2231‐A‐30‐3. Both, the transmitted light and the out‐coming light from the edges were collected with an AvaSphere 30‐Irrad Integrated sphere coupled to an Avantes Spectrometer 2048L. The light was collected on three adjacent spots per edge/surface (5 mm measurement port for AvaSphere 30‐Irrad Integrated sphere). The reported light‐guiding percentage is calculated based on the average of the light collected in the 12 spots measured on the 4 side edges. Finally, solid FP‐LSCs were prepared following the above procedure with the form of a block upon adjusting the FP amount in the initial aqueous solution. The FP liquid‐LSCs were prepared implementing the protein solution to a cuvette containing 3 mL PBS buffer solution. The photophysical characterization (excitation and emission spectra, ϕ, τ) was carried out with a FS5 Spectrofluorometer from Edinburgh Instruments, using the SC‐5 module for liquid samples, the SC‐10 module for solid samples, and the SC‐30 Integrating Sphere to determine ϕ for solution and Quantaurus‐QY Absolute PL quantum yield spectrometer from Hamamastu to determine ϕ for solid. All measurements were performed in ambient conditions. < τ> was calculated by following equation:

(1) $$\langle \tau \rangle =\frac{A_{1}\tau_{1}^{2}+A_{2}\tau_{2}^{2}}{A_{1}\tau_{1}+A_{2}\tau_{2}}$$

A_n_ and τ_n_ are the n‐th amplitude and lifetime parameters obtained from the bi‐exponential fit. The photostability test was carried out by monitoring the ϕ loss as well as changes in the excitation and emission spectra over time either under constant illumination (WINGER WEPW3‐S1 Power LED Star white (6500 K) with a power of 1000 W m^−2^ as pumping source to simulate irradiation; the employed power source was a Keithley 2231‐A‐30‐3) or stored under ambient dark conditions. An average of three independent measurements is presented in this work, showing a deviation of <5%.

### LSC‐Solar Panel Systems Characterization

Both FP liquid‐ and solid‐state LSCs were coupled with a solar panel (a series of 8 monocrystalline Si‐PV cells) IXOLAR SolarBITs (KXOB2‐02×8F; Figure S9, Supporting Information). They were irradiated with solar simulated light (1 sun, 1000 W m^−2^) calibrated with a KG5‐filtered Silicon reference cell (Newport/Oriel, Model 91 150 V) using solar simulator Sol3A 94023A (Class AAA, 450 W Xenon Lamp, AM 1.5G) from Newport for solid LSCs measurement and LSH‐7320 (Class ABA, LED lamp, 0.1 to 1.1 sun adjustable) Newport for liquid LSCs measurement. In the case of applying reflective tapes, aluminum foil were put on four edges including back of solid‐state LSCs. *J–V* curves were recorded using linear sweep voltammetry with PGSTAT30 potentiostat (Ecochemie). The IPCE characterization was carried out using QEPVSI‐b (Newport) and was recorded from 300 to 1100 nm Lasing scan software. All IPCE curves were multiplied by an area factor of 2.8 to consider the illuminated area to the whole surface of LSCs. The device photostability was carried out by monitoring the loss of relative η_opt_ gain with respect to the reference (L‐R and E‐R) over time under continuous irradiation with WINGER WEPW3‐S1 Power LED Star white (6500 K) with a power of 1000 W m^−2^ as pumping source to simulate sun irradiation using the above reference for calibration; the employed power source was a Keithley 2231‐A‐30‐3. An average of three independent measurements is presented in this work, showing a deviation of <4%.

The figures of merit reported in Tables 1 and 2 were calculated as follows:^[^ ^3^ ^]^

(2) $$G=\frac{A_{s}}{A_{e}}=2.9$$

G is the geometry factor, *A_s_* and *A_e_* are the surface area exposed to radiation and the total area of the edged of the LSC;

(3) $$\eta_{opt}=\frac{I_{sc}^{L}A_{e}}{I_{sc}A_{s}}=\frac{I_{sc}^{L}}{I_{sc}G}$$

where η_opt_ is the optical efficiency, $I_{sc\;}^{L}$ represents the short circuit current when the PV is coupled to the LSC and *I_sc_* is the corresponding value for the Si‐cell directly exposed to the simulated solar light—Figure S9 (Supporting Information);

(4) $$J_{sc}=\frac{I_{sc}^{L}}{A_{s}}$$

where J_sc_ is the short‐current density;

(5)

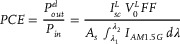

were PCE is the power conversion efficiency, $I_{sc\;}^{L}\;$, $V_{0}^{L}$ and FF is the short circuit current, voltage, and the fill factor of PV when the PV is coupled to the LSC, respectively, AM 1.5G is the standard one sun irradiation;

(6) $$Relative\;gain\;of\;\eta_{opt}=\frac{\eta_{opt\;-protein\;based\;LSC}-\;\eta_{opt-empty\;matrix}\;}{\eta_{opt-empty\;matrix}}\times 100$$

(7) $$Relative\;\;gain\;\;of\;PCE=\frac{PCE_{\;-protein\;based\;LSC}-\;PCE_{-empty\;matrix}\;}{PCE_{-empty\;matrix}}\times 100$$

where η_opt‐protein based LSC_/PCE_‐protein based LSC_ is the η_opt_/PCE of protein based LSCs and η_opt‐empty matrix_/PCE_‐empty matrix_ is the η_opt_/PCE of the LSCs matrix without proteins.

Estimated J_SC_ from integration of IPCE was calculated as following equation:

(8) $$J_{\mathrm{sc}}=q\int_{\lambda_{1}}^{\lambda_{2}}\frac{\mathrm{EQE}\left(\lambda \right)\times \mathrm{AM}1.5G\left(\lambda \right)d\left(\lambda \right)}{\frac{\mathrm{hc}}{\lambda }}$$

where q is the elementary charge, h is the Planck's constant, and c is the speed of light, EQE is the spectrum of IPCE corrected after the area factor.

## Conflict of Interest

The authors declare no conflict of interest.

## Supporting information



Supporting Information

## Data Availability

The data that support the findings of this study are available from the corresponding author upon reasonable request.
