# Supplementary material for: Fluorescent Protein Solid‐State Luminescent Solar Concentrators
Source: Small. 2025 Oct 22;21(48):e07761. doi: 10.1002/smll.202507761 (PMC12674110; doi:10.1002/smll.202507761)
Supplement: Supplementary file 1 — Supporting Information [file SMLL-21-e07761-s001.docx]

Supporting Information

**Fluorescent Protein Solid-State Luminescent Solar Concentrators**

*Sihan Lei, Sara Ferrara, Sanchari Chowdhury, and Ruben D. Costa**

Technical University of Munich, Campus Straubing for Biotechnology and Sustainability, Chair of Biogenic Functional Materials, Schulgasse 22, Straubing 94315, Germany.
E-mail: ruben.costa@tum.de

**Table S1**. Overview of reported LSCs incorporating different types of fluorophores and host matrix.

| Fluorophore types | Fluorophores | Host material | G | Optical efficiency (%)/Optical external efficiecncy | Reference |
| --- | --- | --- | --- | --- | --- |
| Fluorescentm proteins | eGFP | Water/glass container | 2 | 3.30 | ^[34]^ |
|  | mScarlet | Water/PDMS slab container | 0.54 | 2.58 | ^[36]^ |
|  | R-PE | Water/glass container | 2 | 6.88 (with reflective tape) | ^[33]^ |
| Rare earth ions | Eu(III) chelate pigment | waterborne acrylic resins | 5 | 4.5 | ^[14]^ |
|  | Eu(III) | PMMA | - | - | ^[15]^ |
|  | Eu(III) | PMMA | - | 0.68 |  |
| Metal comlex | Eu(TTA)₃phen | PMMA | 5 | 2.47 | ^[17]^ |
|  | di-ureasil–Eu(2mCND)₃·2H₂O hybrid | PMMA | 3.1 | 0.51 | ^[18]^ |
| Quntum dots | CIS/ZnS | PMMA | 6.25 | 1.83 | ^[19]^ |
|  | CdSe/CdS | PDMS | 5 | 7.4 | ^[21]^ |
|  | CISeS | Glass | 3.08 | - | ^[22]^ |
|  | Si | Glass | 15 | 7.9 | ^[27]^ |
| Organic dyes | 3,3′-(2,5-dimethoxy-1,4-phenylene)bis(1-(thiophen-2-yl)prop-2-yn-1-one) | poly(cyclohexyl methacrylate) (PCMA) |  | 7.7 | ^[28]^ |
|  | Lumogen F Red 305 | EVA | 10.69 | - | ^[29]^ |
|  | 4CzTPN-Me | methyl methacrylate (MMA) | 25 | 10.4 (4 PVs) | ^[32]^ |


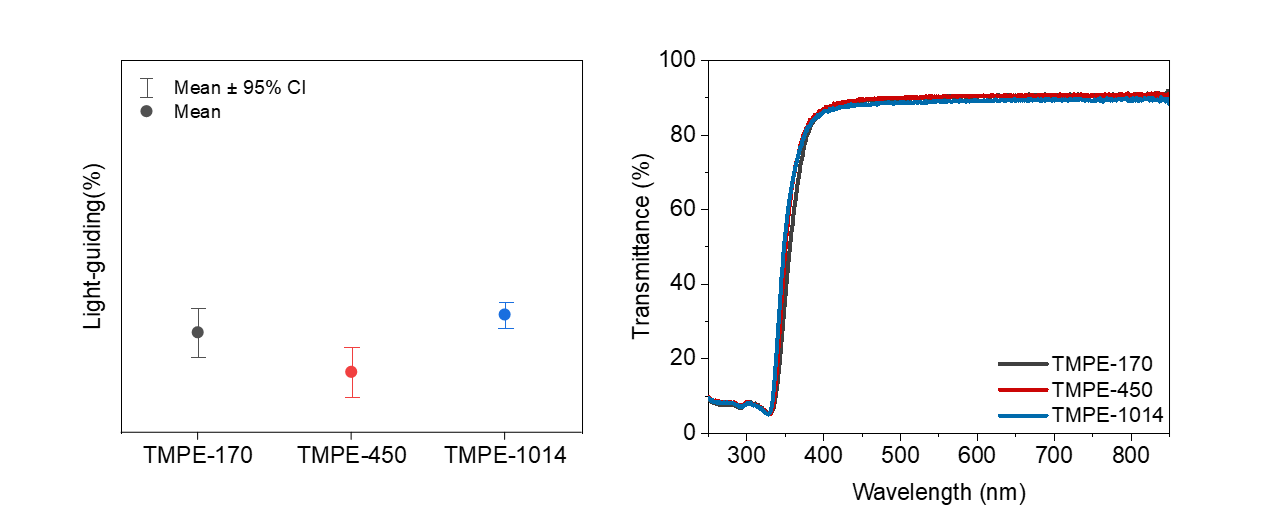


**Figure S1**. Light-guiding (left) and transmittance (right) features of epoxy with different type of TMPE derivatives (see legend).


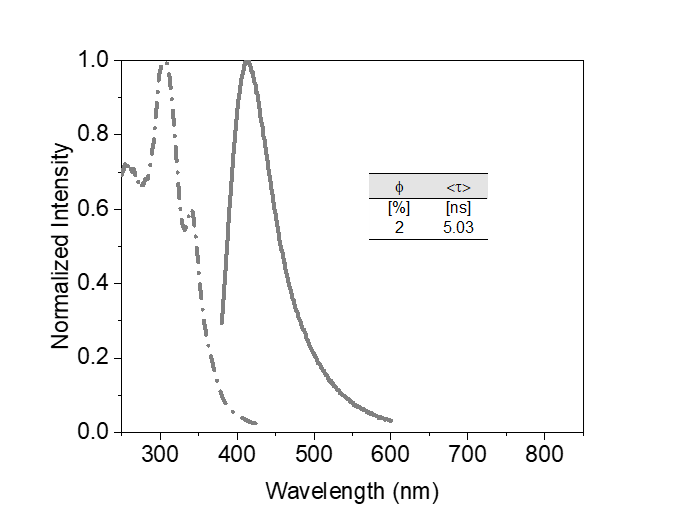


**Figure S2**. Normalized excitation (dashed-dot line) and emission (solid line) spectra of epoxy (50 μL PBS buffer with 0.066 g TMPE-170) with an inset table gathering the figures-of-merit of ϕ (λ_ex_ = 400 nm) and <τ> (λ_ex_ = 375 nm).


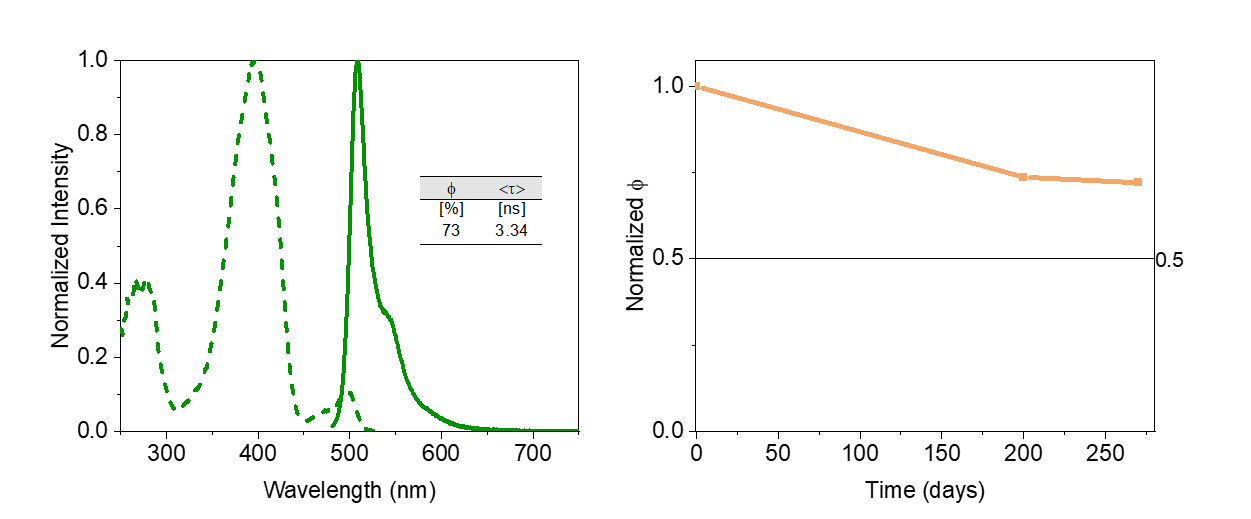


**Figure S3**. Left: Normalized excitation (dashed line) and emission (solid line) spectra of T-Sapphire in PBS buffer and an inset table gathering the figures-of-merit of φ (λ_ex_ = 400 nm) and <τ> (λ_ex_ = 375 nm). Right: Normalized ϕ (λ_ex_ = 400 nm) loss over time of 0.5 mg T-Sapphire-TMPE-170 epoxy upon storage.

**Table S2.** Excited state lifetime (τ) and photoluminescence quantum yield (φ) values for T-Sapphire-epoxy with three TMPE derivatives over time upon LED simulated one sun irradiation. Bi-exponential functions are used for the fit of the excited state decay, as well as the average lifetime with relative contributions over time.

| **Sample** | **Time** | **τ_1_** | **A1** | **τ_2_** | **A2** | **<τ>** | **φ** |
| --- | --- | --- | --- | --- | --- | --- | --- |
|  | **[day]** | **[ns]** |  | **[ns]** |  | **[ns]** | **[%]** |
| **TMPE-170** | **0** | 2.12 | 2227 | 3.64 | 712 | 2.66 | 68 |
|  | **3** | 1.87 | 2152 | 3.48 | 764 | 2.51 | 47 |
|  | **7** | 1.89 | 2016 | 3.74 | 960 | 2.79 | 38 |
|  | **11** | 1.94 | 1842 | 4.50 | 1013 | 3.38 | 34 |
|  | **27** | 1.81 | 1947 | 4.91 | 971 | 3.59 | 18 |
|  | **38** | 1.61 | 2090 | 4.80 | 1142 | 3.59 | 16 |
|  | **48** | 1.95 | 2035 | 5.54 | 1118 | 4.14 | 10 |
|  | **60** | 1.42 | 1770 | 5.15 | 1344 | 4.16 | 9 |
| **TMPE -450** | **0** | 2.43 | 2120 | 3.8 | 728 | 2.91 | 66 |
|  | **5** | 2.2 | 2308 | 5.09 | 500 | 3.16 | 35 |
|  | **28** | 1.63 | 1812 | 4.89 | 1270 | 3.84 | 16 |
|  | **35** | 1.69 | 1793 | 5.23 | 1273 | 4.12 | 14 |
| **TMPE -1014** | **0** | 2.25 | 2577 | 4.5 | 277 | 2.65 | 60 |
|  | **3** | 1.79 | 2208 | 4.76 | 714 | 3.16 | 19 |
|  | **7** | 1.94 | 2255 | 5.03 | 693 | 3.31 | 13 |
|  | **17** | 1.76 | 2049 | 4.96 | 944 | 3.57 | 9 |
|  | **21** | 1.52 | 1959 | 4.86 | 1066 | 3.64 | 8 |
|  | **25** | 1.36 | 1948 | 4.93 | 1270 | 3.87 | 7 |


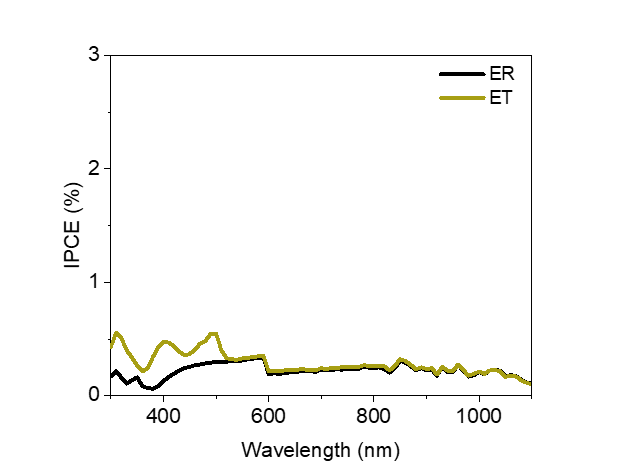


**Figure S4.** IPCE scans of LSCs embedding 0.5 mg T-Sapphire (ET) and reference without protein (ER).


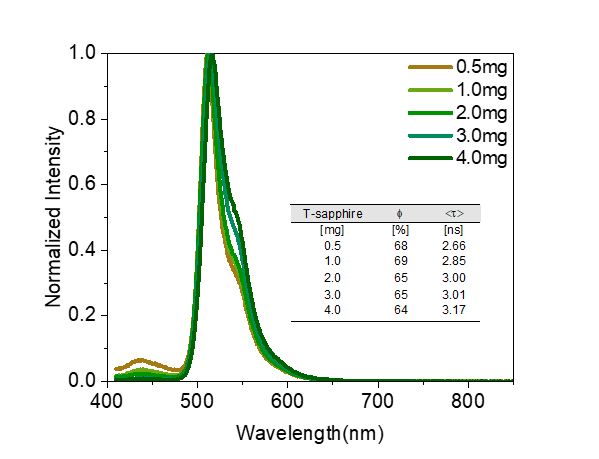


**Figure S5.** Normalized emission (solid line) spectra of increasing amount of T-Sapphire in epoxy with TMPE-170 (see legend). The inset table gathers the figures-of-merit of φ (λ_ex_ = 400 nm) and <τ> (λ_ex_= 375 nm).


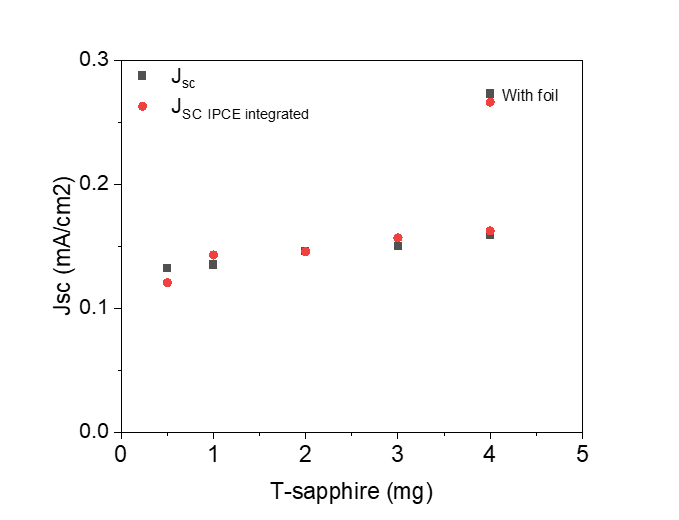


**Figure S6**. Correlation between J_sc_ integrated from IPCE and measured J_sc_ upon increasing amount of T-Sapphire in FP-solid LSCs (see legend). Those named “with foil” refer to LSCs with 4.0 mg T-Sapphire-TMPE-170 epoxy using four reflective tapes.


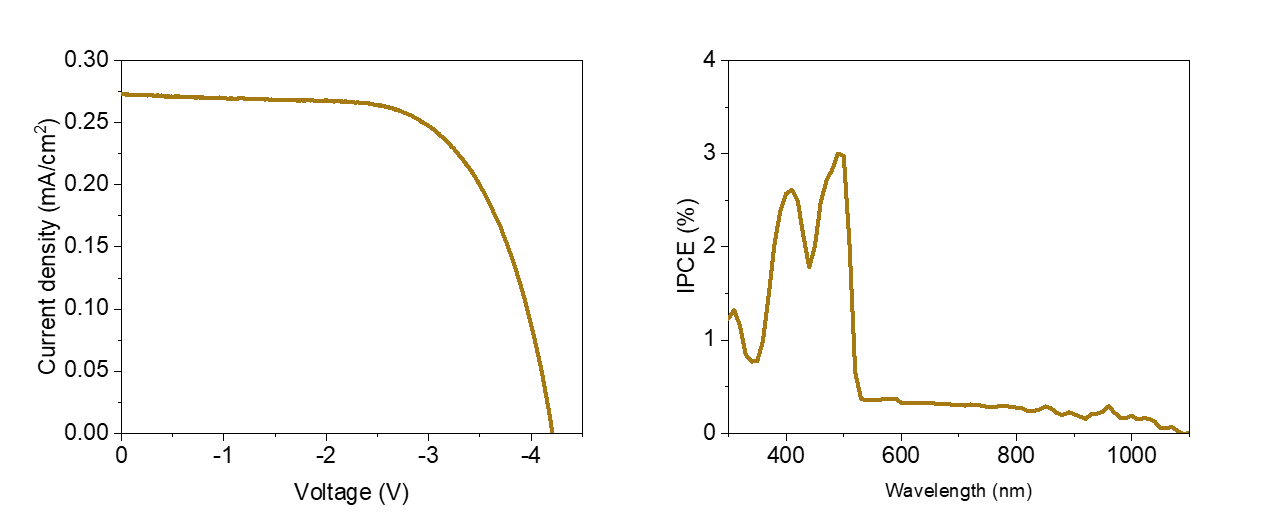


**Figure S7.** Current density-voltage (left) and IPCE (right) of LSCs with 4.0 mg T-Sapphire-TMPE-170 epoxy using four reflective tapes.


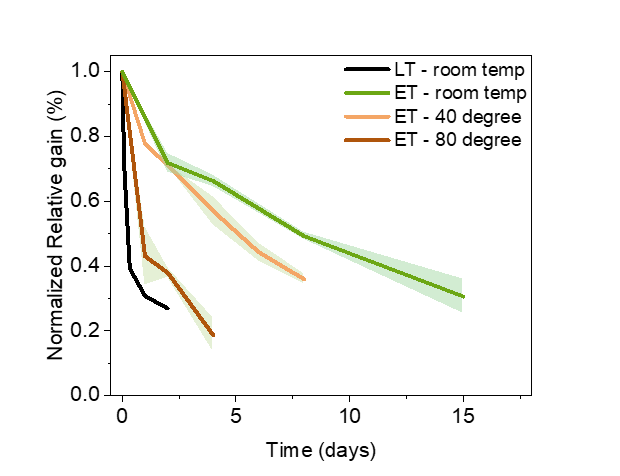


**Figure S8.** Normalized loss of relative η_opt_ gain of solution (LT) and epoxy (ET) T-Sapphire (0.5 mg) LSCs with different thermal stress (see legend) under white LED simulated one sun irradiation over time. The shadow region indicates the standard deviation of 3 samples.


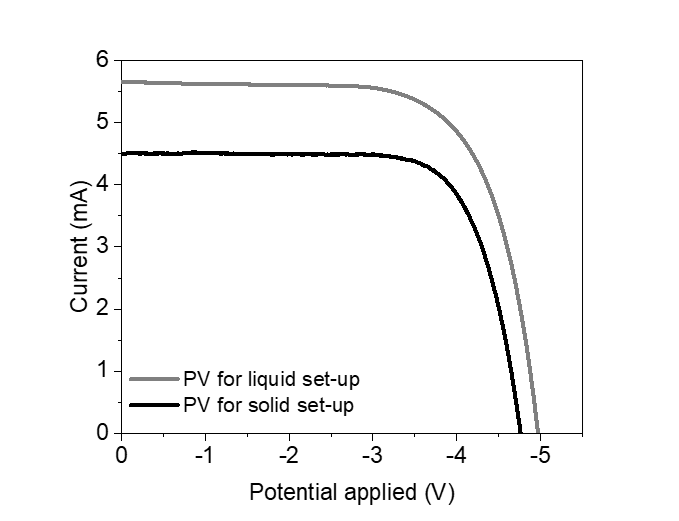


**Figure S9.** Current-voltage of the two respective independent Si-PV solar panels (a series of 8 monocrystalline Si-PV cells) used in this work for solid-state (dark grey) and liquid (light grey) LSCs under 1 sun AM1.5G irradiation.
